# Sex-Differences in Oral Anticoagulant-Related Intracerebral Hemorrhage

**DOI:** 10.3389/fneur.2022.832903

**Published:** 2022-03-03

**Authors:** Josefine Grundtvig, Christian Ovesen, Thorsten Steiner, Cheryl Carcel, David Gaist, Louisa Christensen, Jacob Marstrand, Per Meden, Sverre Rosenbaum, Helle K. Iversen, Christina Kruuse, Thomas Christensen, Karen Ægidius, Inger Havsteen, Hanne Christensen

**Affiliations:** ^1^Department of Neurology, Bispebjerg Hospital, Copenhagen, Denmark; ^2^Department of Clinical Medicine, University of Copenhagen, Copenhagen, Denmark; ^3^Department of Neurology, Klinikum Frankfurt Höchst, Frankfurt, Germany; ^4^Department of Neurology, Heidelberg University Hospital, Heidelberg, Germany; ^5^The George Institute for Global Health, University of New South Wales, Sydney, NSW, Australia; ^6^Research Unit for Neurology, Odense University Hospital, University of Southern Denmark, Odense, Denmark; ^7^Department of Neurology, Rigshospitalet, Copenhagen, Denmark; ^8^Department of Neurology, Herlev Hospital, Copenhagen, Denmark; ^9^Department of Radiology, Bispebjerg Hospital, Copenhagen, Denmark

**Keywords:** stroke, sex-differences, ICH, oral anticoagulation, vitamin K-antagonist, stroke in women, intracerebral hemorrhage (ICH), NOAC

## Abstract

**Introduction and Aim:**

Data remain limited on sex-differences in patients with oral anticoagulant (OAC)-related intracerebral hemorrhage (ICH). We aim to explore similarities and differences in risk factors, acute presentation, treatments, and outcome in men and women admitted with OAC-related ICH.

**Method:**

This study was a retrospective observational study based on 401 consecutive patients with OAC-related ICH admitted within 24 h of symptom onset. The study was registered on osf.io. We performed logarithmic regression and cox-regression adjusting for age, hematoma volume, Charlson Comorbidity Index (CCI), and pre-stroke modified Ranking Scale (mRS). Gender and age were excluded from CHA_2_DS_2_-VASc and CCI was not adjusted for age.

**Results:**

A total of 226 men and 175 women were identified. More men were pre-treated with vitamin K-antagonists (73.5% men *vs*. 60.6% women) and more women with non-vitamin K-antagonist oral anticoagulants (26.5% men *vs*. 39.4% women), *p* = 0.009. Women were older (mean age 81.9 *vs*. 76.9 years, *p* < 0.001). CHA_2_DS_2_-VASc and CCI were similar in men and women.

Hematoma volumes (22.1 ml in men and 19.1 ml in women) and National Institute of Health Stroke Scale (NIHSS) scores (13 *vs*. 13) were not statistically different, while median Glasgow Coma Scale (GCS) was lower in women, (14 [8;15] *vs*. 14 [10;15] *p* = 0.003).

Women's probability of receiving reversal agents was significantly lower (adjusted odds ratio [*aOR*] = 0.52, *p* = 0.007) but not for surgical clot removal (*aOR* = 0.56, *p* = 0.25). Women had higher odds of receiving do-not-resuscitate (DNR) orders within a week (*aOR* = 1.67, *p* = 0.04). There were no sex-differences in neurological deterioration (*aOR* = 1.48, *p* = 0.10), ability to walk at 3 months (*aOR* = 0.69, *p* = 0.21) or 1-year mortality (adjusted hazard ratio = 1.18, *p* = 0.27).

**Conclusion:**

Significant sex-differences were observed in age, risk factors, access to treatment, and DNRs while no significant differences were observed in comorbidity burden, stroke severity, or hematoma volume. Outcomes, such as adjusted mortality, ability to walk, and neurological deterioration, were comparable. This study supports the presence of sex-differences in risk factors and care but not in presentation and outcomes.

## Introduction

There is a growing literature documenting sex differences in risk factors, presentation, treatment interventions, and outcome in stroke. Women are in general older at the time of stroke and have a higher frequency of hypertension (1). Atrial fibrillation (AF) increases the risk of stroke more in women than it does in men (2). Women are also more likely to present with non-traditional stroke symptoms as compared to men and women possibly have a greater benefit from physical activity in the prevention of cardiovascular disease (1). It is consistently reported that women have worse outcomes after stroke, which partly results from older age, worse pre-stroke functional status, and higher comorbidity; however, after adjusting for these factors, women still have worse outcomes (1).

A recent publication demonstrated (3) that women with acute stroke were more likely to be admitted to an acute stroke unit, but less likely to be intubated, to be given treatment for fever, or be admitted to an Intensive Care Unit (ICU). On admission, women had higher odds of having received pre-stroke antihypertensive medication and lower odds of taking antiplatelets, being treated with antidiabetics or lipid-lowering drugs, while no differences in the use of oral anticoagulants were observed. In the total stroke population, case-fatality rates were higher in women than men. However, in multivariable analyses, the risk of death was lower in women than men indicating that the unadjusted estimates were due to confounding. This finding was not significant in the intracerebral hemorrhage (ICH) population alone (3). Based on the 5-dimensional EuroQol, female patients have significantly worse scores on all parameters except for mobility (3). A sub-analysis of the Intensive Blood Pressure Reduction in Acute Cerebral Hamorrhage trials (INTERACT 1 and 2) found a higher adjusted mortality in men as compared to women (4).

Oral anticoagulant (OAC)-related ICH is a devastating presentation of stroke with very high fatality rates (5–7). Following the increased use of OAC, the number of patients with OAC-related ICH is increasing (8). So far, there are no reports available that have explored if sex-differences are present in the OAC-related ICH.

The aim of this study was to investigate that sex differences were present in risk factors, acute presentation, treatment interventions, and outcome in OAC-related ICH.

## Materials and Methods

### COOL-ICH

**Figure 1:**
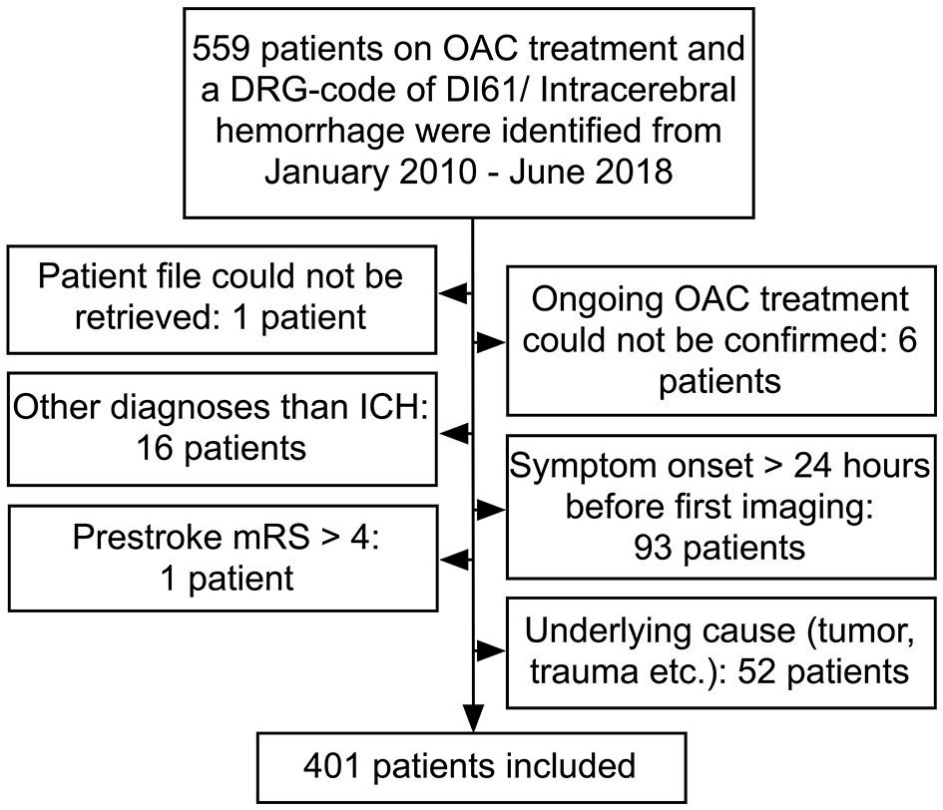
Patient flow. Patients may be counted more than once, if they fulfilled more than one exclusion criteria. OAC, oral anticoagulant; ICH, intracerebral hemorrhage; mRS, modified Rankin Scale.

The analysis was performed using data from the Capital Region Anticoagulation-related ICH study (the COOL-ICH study) cohort. The cohort was based on all patients presenting with an imaging-confirmed OAC-related ICH within 24 h of symptom onset to one of five neurological or neurosurgical departments in the Capital Region of Denmark (approximate population of 1.9 million) from January 2010 to June 2018. In Denmark, the procedure for all patients with stroke is to admit them to a stroke unit and these admissions are then audited by the Danish Stroke Registry (9, 10). For this study, patients were identified for the COOL-ICH cohort using two different approaches: (1) the Danish Stroke Registry in which all departments treating stroke patients are legally obliged to report to and (2) by discharge lists from the individual neurology and neurosurgery departments. Patients with an underlying cause (e.g., vascular malformation or trauma) or a pre-stroke modified Rankin Scale (mRS) above 4 were excluded (Figure 1).

National Institute of Health Stroke Scale (NIHSS) was if not available in patients' files estimated (11). Female sex and age were excluded from CHA_2_DS_2_-VASc (12) as a predictor to be able to compare men and women. To be able to assess multimorbidity separate from age, we did not age-adjust Charlson Comorbidity Index (CCI) (13). The ability to walk at 3 months was assessed based on the note closest to 3 months after stroke in the patient's file.

All clinical imaging was systematically assessed by one senior neuroradiologist (IH). The neuroradiologist was blinded to all other information than the imaging prescription note. This would always include sex and age and may or may not have included information on OAC and concomitant diseases. Hematoma volume was assessed using the ABC/2 method (14). For each participant outcome, data (neurological deterioration and ability to walk) were adjudicated by two independent senior neurologists (LC, JM, PM, SR, HI, CK, TC, and KÆ) based on medical charts, which were blinded for admission to hospital, treating physicians and type of oral anticoagulant; in cases of disagreement, HC had the final say. Neurological deterioration was defined as a fall in Glasgow Coma Scale (GCS) of 2 points or more, and an increase in NIHSS of 4 points or more or 4 strokes. In progression (SIP) score points, using the first recorded neurological status as reference. A more comprehensive protocol is available online, which was completed before the initiation of data acquisition (15).

### MEDSTAT

MEDSTAT is a publicly available online database based on all prescription drugs dispensed at Danish pharmacies, from which aggregate yearly numbers can be retrieved (16). Based on numbers from the Capital Region of Denmark, accessed in MEDSTAT, we retrieved the number of patients for each year (2010–2018) who had presented a prescription for a Vitamin-K antagonist [VKA, ATC B01AA (17)], Dabigatran [ATC B01AE07 (18)], or a direct factor Xa inhibitor [ATC B01AF (18)].

### Statistical Analysis

For each group (men or women), we calculated means and medians as appropriate on numerical variables and percentages for categorical variables. Differences were tested using the *t*-test or Mann–Whitney *U* test and the chi-square or Fisher's exact test. Based on the MEDSTAT data, we made a figure, including the number of patients per year of both men and women treated with either VKAs or non-vitamin K-antagonist oral anticoagulants (NOACs) during 2010–2018, as well as a figure with number of ICHs per year, per 1,000 patients who had presented at least one prescription for either NOAC or VKA during that year. We calculated unadjusted odds ratios (ORs) between men and women for pharmacological reversal, surgical clot removal, external ventricular drains (EVD), ICU (neuro-intensive or general), intubation, and do-not-resuscitate (DNR) orders within 24 h as well as within the first week, neurological deterioration within 24 h and 7 days and finally for the ability to walk at 3 months. We also calculated adjusted ORs for men and women with the above-mentioned variables; adjusting for age, CCI, hematoma volume, pre-stroke mRS, and admission GCS. The Cox regression was performed using days from ICH admission until both 1-year death and 7-day death with sex as the independent variable. Both an unadjusted hazard ratio (HR) was calculated as well as one where we adjusted for the same variables as in the logistic regression. For pre-stroke mRS, we detected non-proportional hazards, and stratified this variable, with only a small change in the coefficient for sex.

### Ethics

The COOL-ICH study was approved by the Danish Patient Safety Authority (3-3013-2102/1) and the Danish Protection Agency (2012-58-0004). No ethical approval was needed for this study according to the Danish law. Data reporting was followed the STROBE statement.

## Results

### Occurrence of OAC-ICH in Men and Women

**Figure 2:**
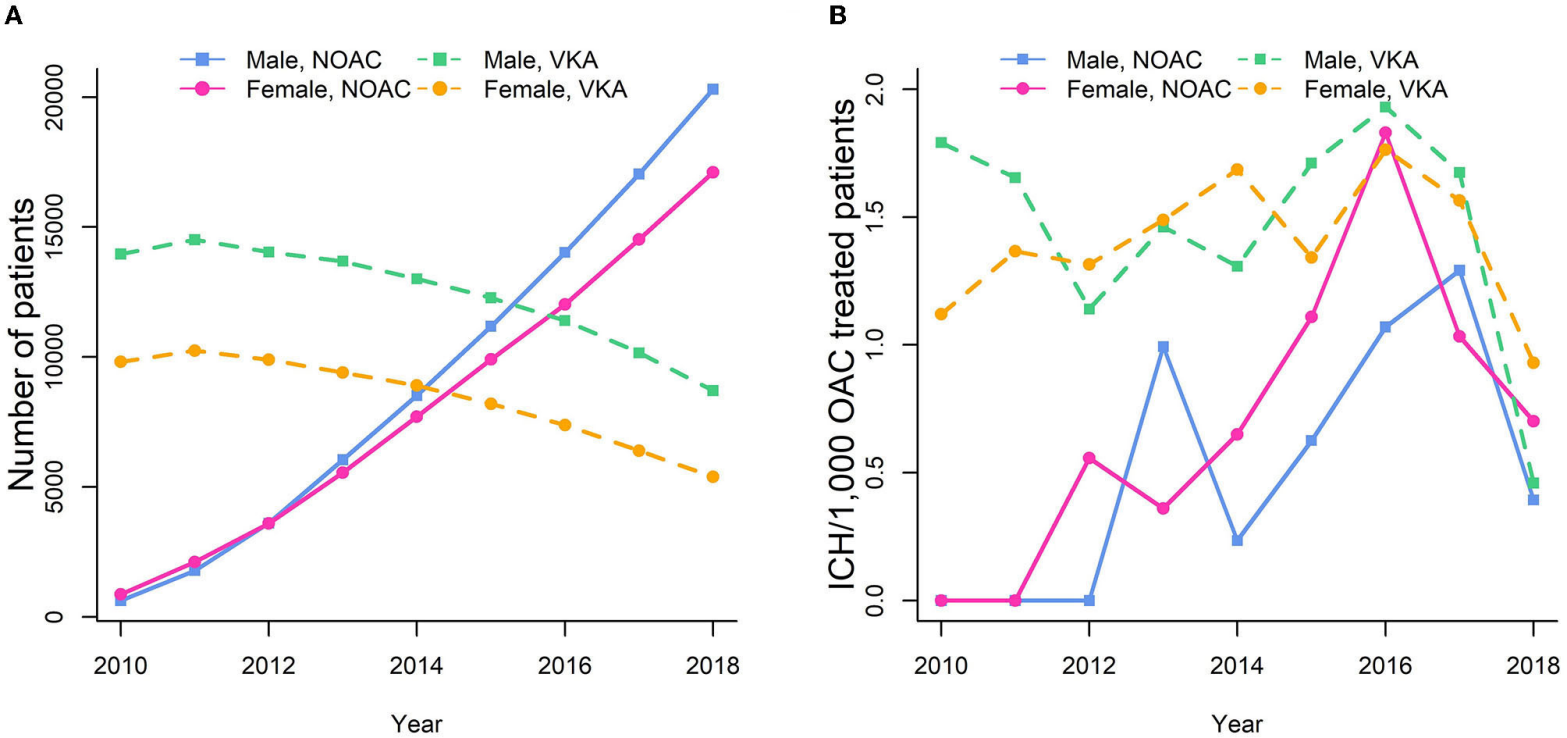
**(A)** Sale of OAC by sex. **(B)** ICHs/1,000 patients who have presented at least one prescription within that year.

In the COOL-ICH cohort, 226 (56.4%) patients were men and 175 (43.6%) patients were women. Looking at sales of OAC, more men than women were treated with both NOAC and VKA during the study period, but number of ICH per 1,000 patients treated with OAC were comparable, though most years, this was a little lower for NOAC-treated men than for NOAC-treated women (Figure 2).

### Patient Characteristics

**Figure 3:**
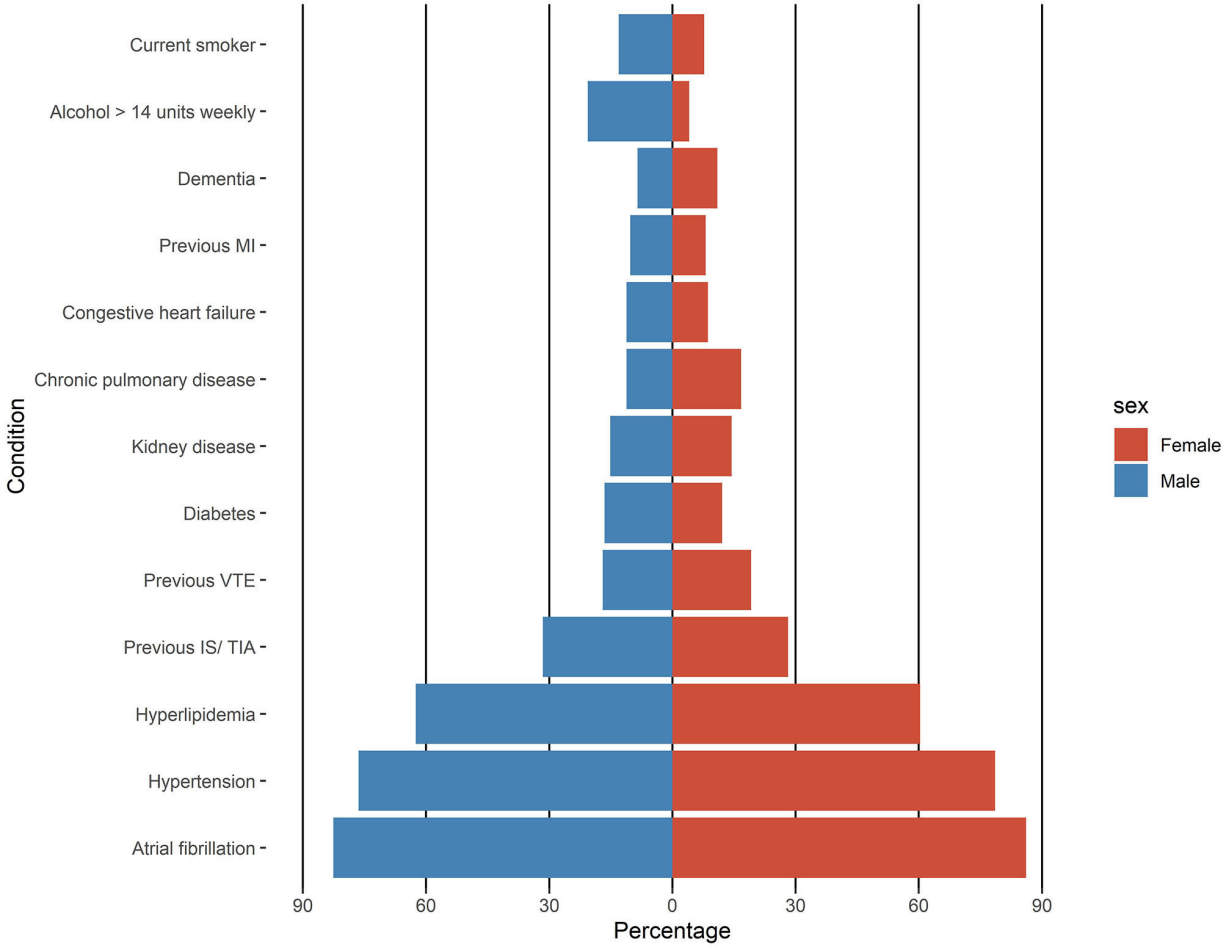
Baseline risk factors in men and women with oral anticoagulant-related intracerebral hemorrhage. MI, myocardial infarction; VTE, venous thromboembolism; IS, ischemic stroke; TIA, transient ischemic attack.

**Figure 4:**
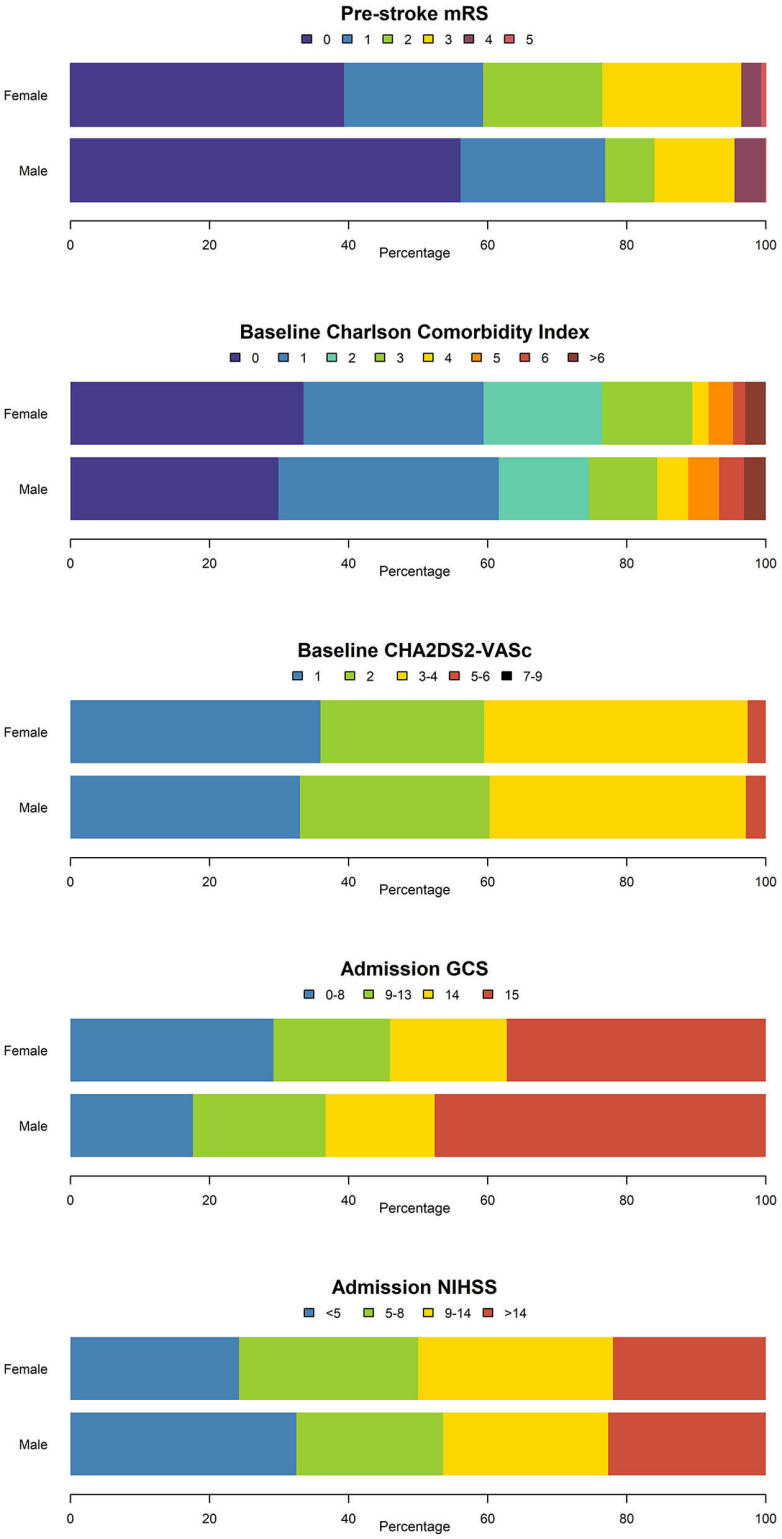
Disability-, comorbidity-, and stroke-severity scores in men and women with OAC-ICH. mRS, modified Rankin Scale; GCS, Glasgow Coma Scale; NIHSS, National Institutes of Health Stroke Scale.

Female patients were significantly older and had a significantly higher pre-stroke mRS (Table 1). No differences were observed in risk factors (AF, hypertension, hyperlipidemia, previous ischemic stroke or transient ischemic attack, previous venous thromboembolism, diabetes, kidney disease, chronic pulmonary disease, congestive heart failure, previous myocardial infarction, and dementia) except for tobacco and alcohol use, where a significant male preponderance was observed (Table 1; Figure 3). There were no differences between men and women in CCI and CHA_2_DS_2_-VASc (Table 1; Figure 4).

**Table 1:** Baseline data in 401 men and women with oral anticoagulant (OAC)-related intracerebral hemorrhage (ICH).

	**Male**	**Female**	***P*-value**
	**(*n* = 226)**	**(*n* = 175)**	
Mean age (SD)	76.9 (8.3)	81.9 (7.4)	<0.001
Modified Rankin Scale	0 (0; 1)	1 (0; 2)	<0.001
Comorbidity scores			
Median Charlson Comorbidity Index (IQR)	1 (0; 3)	1 (0; 2)	0.62
Median CHA_2_DS_2_-VASc (IQR)	2 (1; 3)	2 (1; 3)	0.74
Medicine			
NOAC	60 (26.5%)	69 (39.4%)	0.009
VKA	166 (73.5%)	106 (60.6%)	
Antiplatelets	40 (18.6%)	16 (9.9%)	0.03
Antihypertensives	156 (71.9%)	123 (75.9%)	0.44
Lipid lowering agents	94 (43.9%)	48 (29.6%)	0.006
Selective serotonin reuptake inhibitors	14 (6.5%)	20 (12.3%)	0.08
Smoking status			
Active smoker	22 (13.1%)	10 (7.8%)	<0.001
Former smoker	80 (47.6%)	37 (28.7%)	
Never smoker	66 (39.3%)	82 (63.6%)	
Alcohol/ drug use			
No alcohol use	42 (25.5%)	54 (44.3%)	<0.001
Below 14 units per week	89 (53.9%)	63 (51.6%)	
Above 14 units per week	34 (20.6%)	5 (4.1%)	
Alcohol dependency	20 (8.8%)	2 (1.1%)	0.002
Stroke severity scores			
Admission Glasgow Coma Scale	14 (10; 15)	14 (8; 15)	0.03
Admission NIHSS	8 (4; 13)	10 (5; 15)	0.09
Systolic blood pressure limit order, *n* (%)^a^			
<140 mmHg	53 (23.5%)	42 (24.0%)	0.99
140–160 mmHg	27 (11.9%)	21 (12.0%)	
160–180 mmHg	4 (1.8%)	2 (1.1%)	
No limit ordered	142 (62.8%)	110 (62.9%)	
Radiology			
Median hematoma volume, mL (IQR)	22.2 (4.6; 64.6)	19.1 (4.6; 63.8)	0.90

*OAC, oral anticoagulant; ICH, intracerebral hemorrhage; SD, standard deviation; IQR, interquartile range; NIHSS, National Institute of Health Stroke Scale; mmHg, millimeter of mercury*.

a *Number of patients with a systolic blood pressure limit order in the patient file*.

### Baseline Medications

In the COOL-ICH cohort, fewer men than women were on NOACs and more men were on VKAs (Table 1). Men were more frequently co-treated with antiplatelets as well as lipid-lowering drugs. There were no significant differences between men and women in the use of antihypertensive drugs or selective serotonin reuptake inhibitors (SSRI) (Table 1). Only three patients were concomitantly on dual antiplatelet treatment, one man and two women.

### Acute ICH Event: Presentation and Interventions

Time from ICH symptom onset to admission was similar (*p* = 0.56) for male patients (median: 3.2 h, *IQR*: 1.4; 9.2) and female ones (median: 3.7 h, *IQR*: 1.6; 8.1). No significant difference in time to imaging was observed. Women had marginally more clinically severe presentations on admission, but this only reached significance in GCS and no differences were observed in hematoma volume (Table 1).

Men were significantly more likely to be treated with pharmacological reversal agents (e.g., prothrombin protein complex [any dose], platelet suspension, fresh frozen plasma, tranexamic acid, specific antidotes, etc.) than women (Table 2).

**Table 2:** Interventions and outcomes in 226 men and 175 women with OAC-related ICH.

	**Unadjusted OR**	**95% CI**	***P*-value**	**Adjusted OR^a^, ^b^**	**95% CI**	***P*-value**
Pharmacological reversal	0.45	0.29; 0.70	<0.001	0.52	0.32; 0.84	0.007
Surgical clot removal	0.36	0.14; 0.83	0.02	0.56	0.20; 1.44	0.25
External ventricular drains	0.78	0.39; 1.52	0.47	1.43	0.67; 3.06	0.35
Intensive care unit (neuro or general)	0.83	0.52; 1.33	0.44	1.51	0.87; 2.62	0.14
Intubation	0.95	0.58; 1.54	0.83	1.68	0.94; 3.02	0.08
DNR within 24 h	2.13	1.42; 3.21	<0.001	1.91	1.15; 3.18	0.01
DNR within first week	1.92	1.28; 2.88	0.002	1.67	1.01; 2.77	0.04
Neurological deterioration within 24 h^d^	1.39	0.60; 3.35	0.45	1.93	0.78; 5.07	0.17
Neurological deterioration within 7 days	1.32	0.88; 1.97	0.18	1.48	0.93; 2.38	0.10
Able to walk independently at 3 months	0.58	0.36; 0.93	0.02	0.69	0.38; 1.22	0.21
	**Unadjusted HR**	**95% CI**	***P*** **-value**	**Adjusted HR^a^**	**95% CI**	***P*** **-value**
One-year mortality	1.48	1.14; 1.93	0.003	1.18	0.88; 1.57	0.27

*Odds ratios and hazard ratios for sex, male sex being the reference*.

*OR, odds ratio; CI, confidence interval; HR, hazard ratio; DNR, Do-not-resuscitate order*.

a *Adjusted for age, Charlson Comorbidity Index (CCI), hematoma volume and pre-stroke modified Rankin Scale (mRS)*.

b *7 patients were omitted from the analysis due to missing data*.

c *Based on patients with neurological deterioration within 24 h opposed to within one week*.

No sex differences were present in prescribing systolic blood pressure limits for the administration of acute antihypertensive treatment; however, blood pressure orders were only applied in less than half of the patients (Table 1).

More men than women had surgical clot removal, but this was not significant in the adjusted analysis. No differences were observed in use of EVD (Table 2).

### Do-Not-Resuscitate Orders

**Figure 5:**
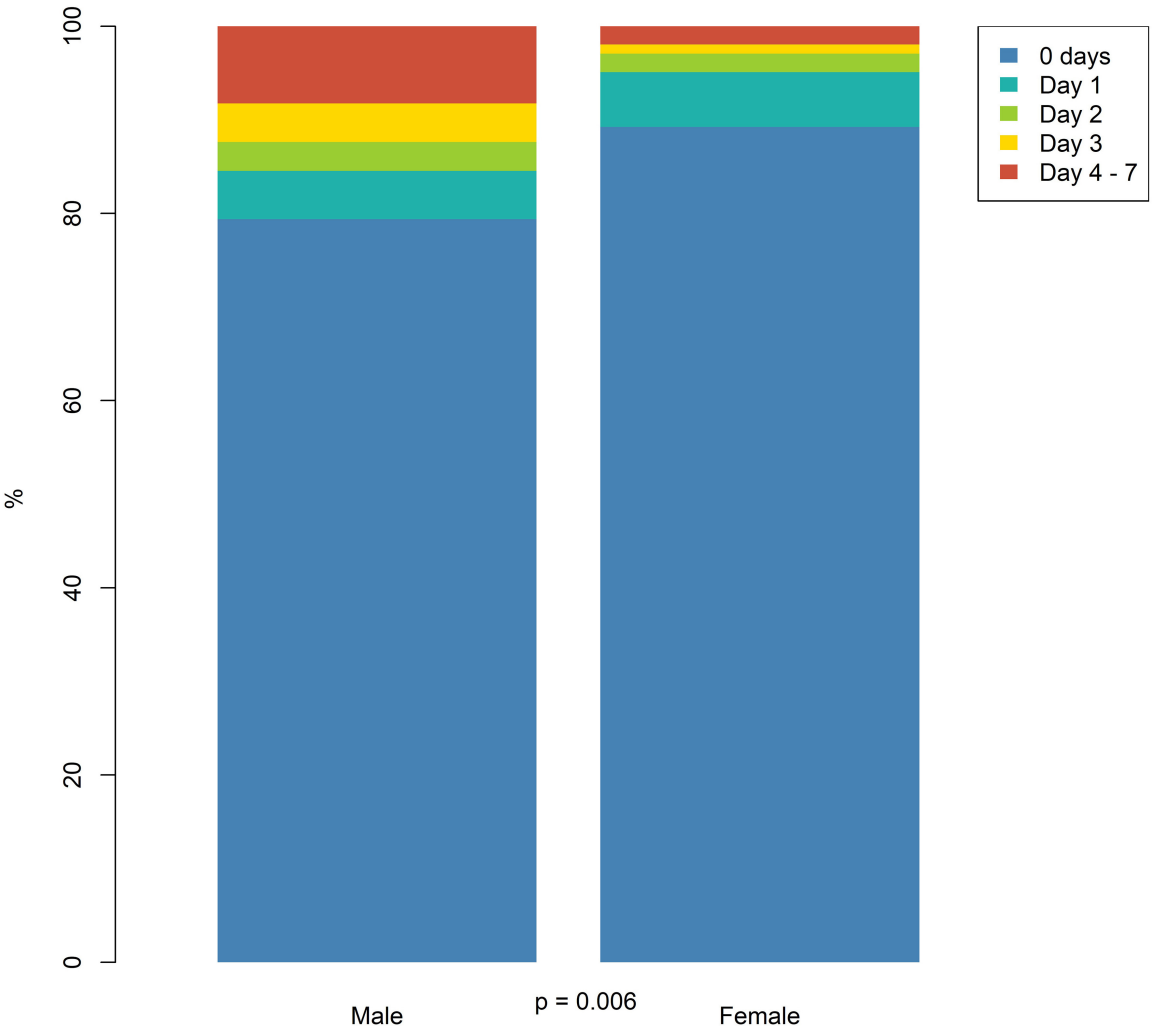
Time from admission to do-not-resuscitate (DNR) within the first week.

Do-not-resuscitate orders (DNRs) were given more often to women both within 7 days and 24 h based on both unadjusted OR and adjusted OR. The time from admission to the DNR decision was significantly longer for men than women, *p* = 0.01 (Figure 5). Of the patients that were given a DNR within 24 h, female patients were significantly older, had a significantly lower CCI as well as significantly lower hematoma volumes (57.6 ml *vs*. 50.2 ml, *p* < 0.001), with no differences between men and women with DNR orders in mRS, GCS, NIHSS, or 1-year mortality (Table 3).

**Table 3:** Patients with OAC-related ICH given DNR orders within 24 h.

	**Male**,	**Female**,	***P*-value**
	***n* = 76**	***n* = 91**	
Age (SD)	78.5 (± 8.3)	83.1 (± 7.9)	<0.001
Charlson comorbidity index	2 (1; 4)	1 (0; 2)	0.009
Pre-stroke mRS	1.0 (0.0; 2.3)	1.0 (0.0; 3.0)	0.09
Admission GCS	9 (6; 13)	9 (5; 14)	0.92
Admission NIHSS	13 (8; 16)	13 (8; 15)	0.88
Hematoma volume	57.6 (27.6; 118.6)	50.2 (15.1; 111.1)	<0.001
One-year mortality	70 (92.1%)	86 (94.5%)	0.76

*OAC, oral anticoagulants; ICH, intracerebral hemorrhage; DNR, do-not-resuscitate; SD, standard deviation; mRS, modified Rankin Scale; GCS, Glasgow Coma Scale; NIHSS, National Institutes of Health Stroke Scale*.

### Outcome

**Figure 6:**
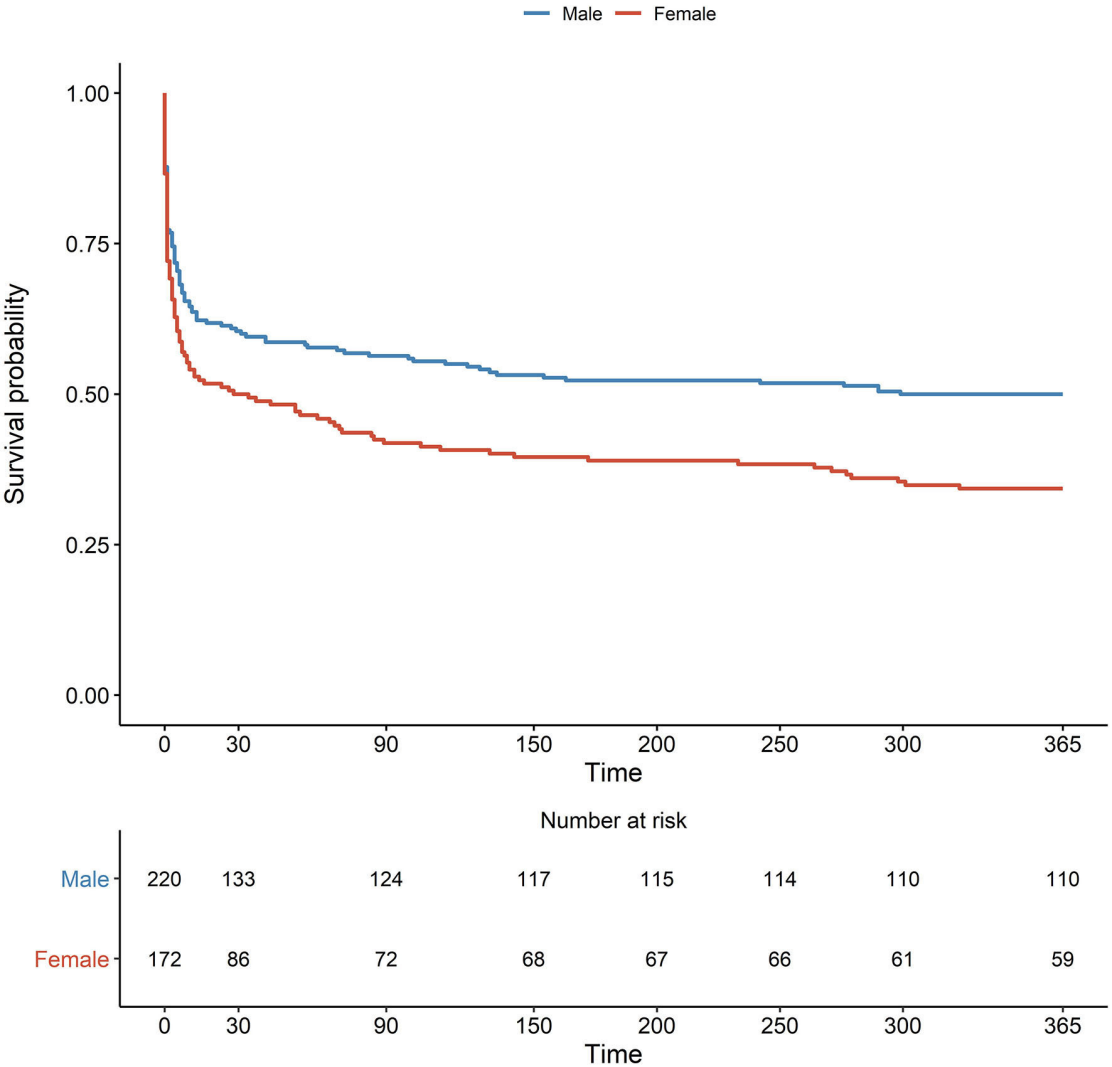
Kaplan–Meier plot with numbers at risk and number of events.

No differences were observed in rates of neurological deterioration. Male patients were significantly more likely to be able to walk at 3 months than female patients, but this was not the case in the adjusted analysis (Table 2). The median time from the admission to the evaluation of the ability to walk was 82 days for men (*IQR*: 42; 92) and 71 days for women (*IQR*: 41; 93), *p* = 0.57. Fifty percent of male patients had died within 1 year of the ICH, in comparison to 65% of women (Figure 6); however, in the Cox-regression analysis, female sex did not increase the risk of 1-year death significantly (*p* = 0.33) when adjusting for age, hematoma volume, CCI, and pre-stroke mRS (Table 2).

## Discussion

More men than women were exposed to OAC in the Capital Region of Denmark during the observation period, with a comparable number of OAC-related ICH per 1,000 OAC-treated patients between men and women each year. Female patients were older and had a higher pre-stroke mRS. CCI and CHA_2_DS_2_-VASc were comparable in men and women. Fewer men than women were pre-treated with a NOAC. More men than women were pre-treated with VKA as well as concomitantly with antiplatelets and lipid-lowering agents. Male patients also had a higher tendency toward smoking and alcohol.

Upon the admission, female patients had a marginally, but significantly, lower GCS, with no differences in NIHSS or hematoma volume. Female patients were less likely to be given pharmacological reversal and more likely to be given a DNR order. When adjusting for age, hematoma volume, pre-stroke mRS, and CCI, there were no differences between men and women in neurological deterioration, ability to walk at 3 months or death.

Data from the American PINNACLE registry showed increased use of VKA in male as compared to female AF patients (19). Another study found that significantly more newly diagnosed male AF patients were treated with almost all types of OAC except for rivaroxaban. They also found a higher rate of ICH in the female patients, though this was no longer significant when adjusting for age, CCI, congestive heart failure, diabetes mellitus, region, insurance plan, and receipt of concomitant medications (20).

The observed differences in risk factors between the sexes, such as age, corroborate previous findings (1, 21). These findings are most likely determined both by biological and cultural factors.

The higher rates of the use of lipid-lowering agents and antiplatelets are in accordance to previous reports (3, 22–26). A potential explanation may be the higher frequency of atherosclerotic disease in men (22–26).

No differences were observed in NIHSS or hematoma volume, albeit GCS was somewhat lower in women. Nonetheless, women less often received reversal agents or clot removal surgery and DNR orders were more frequent. The lower frequency of women treated with clot removal surgery may be explained by the age, CCI, hematoma volume, and pre-stroke mRS of the women (Table 2). These factors, however, did not explain the difference in reversal agents or DNR orders. The sex difference in the use of DNR orders corresponds to previous findings in ICH patients (27) as well as patients successfully resuscitated from in-hospital cardiac arrest (28). The finding of female sex being an independent predictor of receiving a DNR order in the adjusted analyses underlines the need for further investigation into the unconscious bias in the doctors prescribing the DNR orders.

Female sex was no longer an independent predictor of neurological deterioration or inability to walk when adjusting for age, hematoma volume, CCI, and pre-stroke mRS. The observed unadjusted higher mortality in women was probably due to the sex differences in baseline characteristics, as adjusted analysis indicated no sex difference in case fatality.

Despite equal hematoma volume and NIHSS on admission, reversal therapy and surgical hematoma removal were less often applied to women in comparison to men, though in adjusted analysis, this only remained significant for reversal therapy, potentially due to less numbers in surgical treatment. This finding is in line with DNR orders being issued more frequently and earlier in female patients.

Sex differences in stroke care have previously been reported, however, adjusted survival rates are higher in women in comparison to men after stroke in most reports (1, 3, 21, 22, 24). We found no differences between sexes in adjusted 1-year mortality after ICH. However, DNR orders may worsen outcomes independently in ICH patients (29–32) and it is possible that the increased use of DNR orders in women in our study may have contributed to a decreased survival.

Our observation of few interventions in women with ICH is in accordance with previous reports (3). Guidelines in stroke care have changed during the observation period, and recommendations to proactively reverse anticoagulation were only implemented in Denmark at the end of the observation period (33). Nonetheless, this does not explain the difference in reversal attempts between the sexes. Although there is no strong evidence supporting neither reversal therapy so far, this is the case both in men and women, i.e., irrespective of gender (33, 34).

Implicit gender bias has been identified as an explanatory factor in testing in acute myocardial infarction (35), where female sex is associated with frailty and increased caution in relation to interventions. There are so far no data available if this phenomenon is also relevant to stroke.

With the well-known predictive value of DNRs (28–31), our findings underline the need for further investigation into the interaction of age, age bias, sex, and gender bias in treatment decisions. Our findings further support a difference in the pathophysiology of stroke in male and female patients—possibly at least partly based on lifestyle, underlining the need for including women in stroke trials but also the need for research in the pathophysiology of stroke in men and women.

### Limitations

There are some limitations to this study: the study is retrospective and observational and restricted to hospitalized patients. This methodology may have reduced the validity of variables that are mostly narratively described in patients' files, including neurological deterioration and ability to walk at 3-months. Despite mandatory admission to the stroke unit, some patients may have been missed, e.g., due to death before hospital admission or if admitted to another specialty in case of other higher prioritized condition, or if diagnostic brain imaging was only performed at 24 h or later. Based on our data, it was not possible to assess adherence to prescribed OAC. No quality-of-life data were available; these could have provided further insights into qualitative outcomes. As to blood pressure control in the acute phase, we only have data on the prescriptions, not on the actual blood pressure levels.

### Strengths

The study is based on a well-defined catchment area (Capital Region of Denmark) with a public healthcare system providing all acute healthcare for the population free of charge and independent of personal income. The risk of selection bias is, therefore, low. All institutions have easy access to the explored interventions, such as reversal therapies. Data acquisition followed a detailed protocol, which was published before the data collection. All imaging were reassessed confirming a diagnosis, and endpoints adjudicated by senior neurologists.

### Conclusions

This study demonstrated that women receive less treatment interventions and more DNR orders than men after OAC-related ICH. This cannot be fully explained by differences in the presentations of patients though age is no doubt a contributing factor. There is a need for research that can support reducing sex-based inequity in treatment and care. There were no significant differences in ICH rates between men and women.

## Data Availability Statement

The raw data supporting the conclusions of this article will be made available by the authors, without undue reservation.

## Ethics Statement

Ethical review and approval was not required for the study on human participants in accordance with the local legislation and institutional requirements. Written informed consent for participation was not required for this study in accordance with the national legislation and the institutional requirements.

## Author Contributions

CO and HC conceived the study. JG, CO, HC, and TS developed the protocol. HC, JG, CO, TS, and DG planned data analysis. JG, CO, and HC acquired data and performed data analysis. JG performed the statistical analysis. IH planned and performed radiological analysis. LC, JM, PM, SR, HI, CK, TC, and KÆ adjudicated outcomes and HC re-adjudicated outcomes with conflicts between first two observers. All authors reviewed the manuscript critically and approved the final version of the manuscript.

## Funding

This work was supported by the Lundbeck Foundation and Grosserer A.V. Lykfeldt of Hustrus Legat. The funders were not involved in the study design, collection, analysis, interpretation of data, the writing of this article or the decision to submit it for publication.

## Conflict of Interest

CO has received a travel grant from Merck, Sharp, and Dohme. HC has received personal honoraria for speaking/educational activities from Boehringer-Ingelheim, Bristol-Myers-Squibb, Bayer, Daichi-Sanko and has received honoraria paid to her institution for services as National Lead in trials sponsored by Bayer and Alexion. The remaining authors declare that the research was conducted in the absence of any commercial or financial relationships that could be construed as a potential conflict of interest.

## Publisher's Note

All claims expressed in this article are solely those of the authors and do not necessarily represent those of their affiliated organizations, or those of the publisher, the editors and the reviewers. Any product that may be evaluated in this article, or claim that may be made by its manufacturer, is not guaranteed or endorsed by the publisher.
