# Corrigendum: Sex-differences in oral anticoagulant-related intracerebral hemorrhage

**DOI:** 10.3389/fneur.2022.1055360

**Published:** 2022-10-19

**Authors:** Josefine Grundtvig, Christian Ovesen, Thorsten Steiner, Cheryl Carcel, David Gaist, Louisa Christensen, Jacob Marstrand, Per Meden, Sverre Rosenbaum, Helle K. Iversen, Christina Kruuse, Thomas Christensen, Karen Ægidius, Inger Havsteen, Hanne Christensen

**Affiliations:** ^1^Department of Neurology, Bispebjerg Hospital, Copenhagen, Denmark; ^2^Department of Clinical Medicine, University of Copenhagen, Copenhagen, Denmark; ^3^Department of Neurology, Klinikum Frankfurt Höchst, Frankfurt, Germany; ^4^Department of Neurology, Heidelberg University Hospital, Heidelberg, Germany; ^5^The George Institute for Global Health, University of New South Wales, Sydney, NSW, Australia; ^6^Research Unit for Neurology, Odense University Hospital, University of Southern Denmark, Odense, Denmark; ^7^Department of Neurology, Rigshospitalet, Copenhagen, Denmark; ^8^Department of Neurology, Herlev Hospital, Copenhagen, Denmark; ^9^Department of Radiology, Bispebjerg Hospital, Copenhagen, Denmark

**Keywords:** stroke, sex-differences, ICH, oral anticoagulation, vitamin K-antagonist, stroke in women, intracerebral hemorrhage (ICH), NOAC

In the published article, there was an error in Table 1 as published due to erroneous data entry. The reported NIHSS in the table for the male participants is 14 (10; 15) and it is 14 (8; 15) for the female, *p* = 0.88. The correct number for the male participants is 8 (4; 13) and for the female it is 10 (5; 15), *p* = 0.09. The corrected Table 1 and its caption appears below.

**Table 1:** Baseline data in 401 men and women with oral anticoagulant (OAC)-related intracerebral hemorrhage (ICH).

	**Male**	**Female**	***P*-value**
	**(*n* = 226)**	**(*n* = 175)**	
Mean age (SD)	76.9 (8.3)	81.9 (7.4)	<0.001
Modified Rankin Scale	0 (0; 1)	1 (0; 2)	<0.001
Comorbidity scores			
Median Charlson Comorbidity Index (IQR)	1 (0; 3)	1 (0; 2)	0.62
Median CHA_2_DS_2_-VASc (IQR)	2 (1; 3)	2 (1; 3)	0.74
Medicine			
NOAC	60 (26.5 %)	69 (39.4 %)	0.009
VKA	166 (73.5 %)	106 (60.6 %)	
Antiplatelets	40 (18.6 %)	16 (9.9 %)	0.03
Antihypertensives	156 (71.9 %)	123 (75.9 %)	0.44
Lipid lowering agents	94 (43.9 %)	48 (29.6 %)	0.006
Selective serotonin reuptake inhibitors	14 (6.5 %)	20 (12.3 %)	0.08
Smoking status			
Active smoker	22 (13.1 %)	10 (7.8 %)	<0.001
Former smoker	80 (47.6 %)	37 (28.7 %)	
Never smoker	66 (39.3 %)	82 (63.6 %)	
Alcohol/ drug use			
No alcohol use	42 (25.5 %)	54 (44.3 %)	<0.001
Below 14 units per week	89 (53.9 %)	63 (51.6 %)	
Above 14 units per week	34 (20.6 %)	5 (4.1 %)	
Alcohol dependency	20 (8.8 %)	2 (1.1 %)	0.002
Stroke severity scores			
Admission Glasgow Coma Scale	14 (10; 15)	14 (8; 15)	0.03
Admission NIHSS	8 (4; 13)	10 (5; 15)	0.09
Systolic blood pressure limit order, *n* (%)^a^			
<140 mmHg	53 (23.5 %)	42 (24.0 %)	0.99
140–160 mmHg	27 (11.9 %)	21 (12.0 %)	
160–180 mmHg	4 (1.8 %)	2 (1.1 %)	
No limit ordered	142 (62.8 %)	110 (62.9 %)	
Radiology			
Median hematoma volume, mL (IQR)	22.2 (4.6; 64.6)	19.1 (4.6; 63.8)	0.90

OAC, oral anticoagulant; ICH, intracerebral hemorrhage; SD, standard deviation; IQR, interquartile range; NIHSS, National Institute of Health Stroke Scale; mmHg, millimeter of mercury.

a Number of patients with a systolic blood pressure limit order in the patient file.

The authors apologize for this error and state that this does not change the scientific conclusions of the article in any way. The original article has been updated.

## Publisher's note

All claims expressed in this article are solely those of the authors and do not necessarily represent those of their affiliated organizations, or those of the publisher, the editors and the reviewers. Any product that may be evaluated in this article, or claim that may be made by its manufacturer, is not guaranteed or endorsed by the publisher.

